# Dataset on the in-stream and off-stream economic value of water

**DOI:** 10.1016/j.dib.2020.105434

**Published:** 2020-03-16

**Authors:** Benjamin H. Lowe, David R. Oglethorpe, Sonal Choudhary

**Affiliations:** a Sheffield University Management School, Conduit Road, Sheffield S10 1FL, UK; b School of Management, Cranfield University, MK43 0AL, UK

**Keywords:** Benefit transfer, Economic value of water, Environmental valuation, Non-market valuation, Value transfer

## Abstract

This dataset contains 706 estimates of the economic value of water; it has been compiled from published sources. Economic values are provided for three off-stream uses (agriculture/irrigation, industry, and municipal) and three in-stream ecosystem services (recreation, waste assimilation, and wildlife habitat). The dataset covers per period and capitalised asset values. All value estimates have been standardised in USD (2014) per acre-foot. The data accompany the research article entitled “Shifting from volume to economic value in virtual water allocation problems: a proposed new framework and methodology” [1]. The dataset can be used to facilitate benefits (or value) transfer.

Specifications table

**Table:** 

Subject	Economics.
Specific subject area	Environmental economics and, specifically, the practice of environmental valuation (assigning welfare values to goods and services provided by the natural environment).
Type of data	Table
How data were acquired	Compiled from published sources (see description below).
Data format	Raw (converted into a common currency and updated to a uniform moment in time) with descriptive data.
Parameters for data collection	The data consist of standardised estimates of the economic value of water in different contexts. These contexts include off-stream uses (agriculture/irrigation, industry and municipal) and in-stream ecosystem services (recreation, waste assimilation and wildlife habitat). Tailored sub-categories are applied to classify the data in each context. Data include per period and capitalised asset values.
Description of data collection	The data were collected from published environmental valuation studies following a review of five specialist environmental valuation databases. These databases are unstructured and do not conform to a common reporting format. Where possible, the original studies identified in this search were consulted directly to obtain the value estimates included here. The reference sections of those studies that were identified were also consulted to locate additional relevant material.
Data source location	Global data.
Data accessibility	With the article.
Related research article	Lowe, B.H., Oglethorpe, D.R. and Choudhary, S. (2020). Shifting from volume to economic value in virtual water allocation problems: a proposed new framework and methodology. J. Environ. Manage. [In Press].

## Value of the data

• The data contain estimates of the economic value of water in multiple off-stream and in-stream contexts that have been taken from published sources.
• Researchers can use the data to compare the value of water derived from different methods, across different contexts and geographies, and in some cases, across different time periods as approaches to environmental valuation have evolved.
• Researchers can use the data for benefits/value transfer, i.e. the practice of drawing on existing estimates to value environmental goods and services rather than conducting new primary studies.
• Evident gaps in the data may stimulate additional research in the environmental valuation community.

## 1 Data description

The dataset comprises 706 tabulated estimates of the economic value of water in different off-stream and in-stream contexts; these estimates have been taken from 120 published sources. Table 1, Table 2, Table 3 show the distribution of the estimates by context. As shown, the data are split between those estimates that apply to the USA (408) and those that have been estimated in the Rest of the World (ROW) (298). The data include per period values (i.e. represents a single period) (660) and capitalised asset values (i.e. represents the capitalised present value of a stream of future values) (46). All value estimates have been standardised in USD (2014) per acre-foot.

**Table 1:** Classification of USA values according to water category (off-stream).

	Agriculture/Irrigation	Industry	Municipal	Total
No. of per period estimates	209	42	25	276
No. of capitalised asset estimates	12	0	16	28
Total	221	42	41	304

**Table 2:** Classification of ROW values according to water category (off-stream).

	Agriculture/Irrigation	Industry	Municipal	Total
No. of per period estimates	144	89	65	298
No. of capitalised asset estimates	0	0	0	0
Total	144	89	65	298

**Table 3:** Classification of USA values according to water category (in-stream).

Per period values	Recreation	Waste assimilation	Wildlife habitat	Total
No. of per period estimates	49	13	24	86
No. of capitalised asset estimates	0	0	18	18
Total	49	13	42	104

Tables 4 and 5 that follow provide the extended definition of each of the off-stream and in-stream contexts, as well as the detailed assumptions that were used to sub-categorise the agricultural/irrigation water values.

## 2 Experimental design, materials, and methods

### 2.1 Overview

The data were compiled following a review of published environmental valuation studies on the economic value of water (measured in volumetric units) in five specialist environmental valuation databases. These databases were:

• EVRI [2].
• Envalue [3].
• ValueBaseSWE [4].
• The Economics of Ecosystems and Biodiversity Valuation Database [5].
• The New Zealand Non-Market Valuation Database [6].

In addition, the reference sections of those papers identified were searched for additional relevant material. In all cases, the original papers identified in this search were consulted to obtain the original value estimates included here, the exception being where these were no longer available and thus a secondary reference had to be relied upon, provided one was available with sufficient detail.

The water categories/contexts that structured the review of published valuation studies are defined in Table 4.

**Table 4:** Definition of water categories/contexts.

Water category	Definition
Agriculture/Irrigation	Water that is artificially applied during crop cultivation.
Industry	The benefit provided by water used in manufacturing or processing. This might include water for cooling, processing raw materials and general overhead requirements in factories.
Municipal	Water that is used around the home and for commercial (non-industrial) business activities. Domestic water use around the home includes indoor (e.g. for cooking, washing and hygiene) and outdoor (e.g. lawn sprinklers) activities.
Recreation	The benefits provided by direct access to water (e.g. rafting, kayaking and fishing), as well as shoreline based activities (e.g. camping and hiking) which are enriched by proximity to water.
Waste assimilation	The benefit provided by water bodies and rivers that dilute waste and thereby decrease damages that may be suffered by other water users.
Wildlife habitat	The role that water plays in providing a habitat for fish and other wildlife.

Studies were excluded where:

• They were not published in English.
• They referred to one-off unit value estimates for water but with little associated explanation regarding how this estimate was derived.
• They used non-standard volumetric units of measurement (e.g. a bucket of water).
• They did not explicitly derive a unit value estimate but where this may have been feasible with sufficient knowledge of the original study and original context.

With reference to agricultural/irrigation water values specifically, studies were excluded where:

• They had taken a social accounting perspective which looked at what Bernardo et al. [7] call *productivity-related benefits* and which was inconsistent with the private accounting stance adopted across the other water use categories.
• The value had been derived from a ‘gross value’ method that simply divides the value of the crop by the water used to produce it and does not attempt to estimate what *portion* of this value is attributable to water.

With reference to the value of water used in industry, studies have been included which use the added value, cost of intake and residual value approaches. Whilst these approaches have now been superseded [8,9], they have been included here because of the limited number of studies in this area and because these techniques were previously considered appropriate [8]. Therefore, they show how approaches to valuing water in industry have changed over time, but as the magnitude of the resulting values in many cases suggests, the values that these techniques generate should be treated with caution.

It is possible to view the value of water for some recreational activities (such as waterfowl hunting, fishing and angling) as a proxy for the value of water for wildlife habitat. However, in this context, the value of water for wildlife habitat was taken from studies that isolate the value of water for this purpose. This has been achieved either by focusing on:

• Water market transactions that specifically provide water for wildlife habitat.
• Commercial activities (such as commercial fishing) where the value of water does not include a non-commercial or recreational element.

### 2.2 Value standardisation

In line with the approach adopted by other authors who have attempted a similar exercise to this [10], all value estimates have been temporally adjusted to 2014 US Dollars (USD) using the Implicit Price Deflator (IPD) for GDP from the Bureau of Economic Analysis in the USA. Where the valuation year was not explicitly stated in the original study, the date of any underlying data used in the analysis was utilised as a proxy (where this was given as a range, the last year was used), or if this was not possible, the year of publication was used. Where values were denominated in currencies other than USD, the approach advocated by Ready et al. [11] and Czajkowski and Ščasný [12] was utilised. These values were first converted to US Dollars using World Bank Purchasing Power Parity exchange rates for GDP (applicable to appropriate valuation year), then temporally adjusted to 2014 using the IPD.

Where values were given as a simple range (e.g. $10–$20), the median value was used in the standardisation procedure. Where a value was listed as greater than a certain figure (e.g. >$100), then the value given (in this case $100) was used.

Given that the majority of the value estimates were USA specific (nearly 60%), and thus denominated in acre-feet (AF), this was the standardised volumetric measure used to summarise the data to minimise the number of conversions required (1 AF = 1233.48 m^3^).

Many of the sources report value estimates as single monetary figures rather than setting out any marginal relationship, even where marginal relationships exist. Where this abstraction has occurred, the single figure has been labelled as ‘recorded.’ Where the source does provide a fuller picture of a marginal relationship in the form of multiple estimates (e.g. marginal recreation values with differing levels of water flow) then the median value in the range (and the range itself) has been used to ensure that this value is one which is observed. This has been necessary because there are multiple estimates, across different value categories, which have been derived using a variety of different variables, not all of which can be taken into account. Any values included in this way have been labelled as ‘summarised.’ As a result of using a single monetary figure, the assumption of constant returns to scale is implicitly being made in the presentation of value estimates in the dataset.

For each value estimate, the data have been labelled according to the relevant measure of central tendency. For example, if the value has been summarised, then this will be the median value. However, stated preference studies often report *mean* Willingness to Pay as a single figure.

Finally, several sub-categories within each water category have also been defined to classify the data (see Table 3 in Lowe et al. [1]).

The sub-categories were not always explicitly defined in each source. However, sub-categories can be inferred based on the valuation technique employed (e.g. [8,^9^,^13^,14]; 14 provides an easily accessible overview of the principal techniques used). See Table 5 for the assumptions used to sub-categorise the agricultural value estimates.

**Table 5:** Assumptions that were made in the classification of agricultural values (by valuation technique).

Valuation technique	Assumption (unless stated otherwise)
Farm crop budget/residual value	Volumetric measure is applied water. Values are short-run and at site, unless water procurement and fixed costs are explicitly subtracted.
Hedonic Property Value Method	Volumetric measure is withdrawn water. Values are long-run and at site values.
Linear Programming	Volumetric measure is applied water.
Water market transaction	Volumetric measure is withdrawn water. Values are short-run and at site.
Yield comparison	Volumetric measure is applied water. Values are long-run and at site.

The value of agriculture/irrigation water can be defined by the measure of utilisation i.e. the volume of water that is withdrawn or diverted from a water source, that which is applied to the crop, or, that portion of applied water that is consumed during crop growth (sometimes referred to as net irrigation). The value of irrigation water can be further defined in three ways:

• At the source of water extraction or at the site where it is used (depending on whether any costs incurred in extracting the water from the stream and making use of it are included when deriving the water value)
• In the long and short-run (depending on whether or not fixed costs are taken in to account when deriving the water value)
• For high value (or speciality) or low valued crops.

Agricultural crops were classified as either high (e.g. fruit) or low (e.g. small grains) value based on El-Ahry and Gibbons [15]. It should be noted here that this classification, while referring to a generally applicable strata of crop values, came from the USA. Therefore, it was not sufficiently detailed to classify some crops grown in the ROW countries. Where a crop was not classified for this reason, it has been labelled ‘not classified.’ Similarly, where a study was not specific about whether the crop was high or low-value, or where this was unclear, the crop value is referred to as ‘unknown.’ Where summary values are provided for high and low-value crops grown outside of the USA, these should be treated with an element of caution.

The value of water for recreation can be estimated based on variations in water flow. Where this was the case, these flow variations have been recorded, along with the specific recreational activity and the characteristics of the recreational site (e.g. river, reservoir etc.).

### 2.3 Nature of the data and limitations

The value estimates included in the dataset have been calculated using a variety of different methods. These include cost-based techniques that are not based on the demand curve, as well as techniques that derive genuine welfare measures based on Marshallian consumer surplus or the Hicksian compensating or equivalent measures. Related to this, some of the techniques used to generate the value estimates give rise to average values, some give rise to marginal values, and others derive the average value of a marginal increment. Indeed, in some cases, it is not possible to identify what value conception is being identified. Therefore, while this dataset goes some way toward understanding how economic values vary by technique, context and location, not all of the estimates are directly comparable in a strict sense. However, the value estimates have been categorised by technique where possible to address this issue.

## CRediT authorship contribution statement

**Benjamin H. Lowe:** Conceptualization, Methodology, Formal analysis, Investigation, Data curation, Writing - original draft, Writing - review & editing, Visualization. **David R. Oglethorpe:** Conceptualization, Methodology, Writing - review & editing. **Sonal Choudhary:** Conceptualization, Methodology, Writing - review & editing.

## Appendix Supplementary materials

Image, application 1
